# Evaluation of plasma levels of BDNF in patients with disorder depressive

**DOI:** 10.1192/j.eurpsy.2021.898

**Published:** 2021-08-13

**Authors:** D. Galletta, C. Mazzarino, G. Cusumano, A. Santoro

**Affiliations:** Department Of Head-neck Care Unit Of Psychiatry And Psychology “federico Ii” University Hospital Naples, “Federico II” University Hospital Naples, Italy, Naples, Italy

**Keywords:** Depressive Disorder, plasma level, BDNF

## Abstract

**Introduction:**

According to the World Health Organization (WHO, 2017) depressive disorder continues to be the most widespread and growing mental illness in the world, also assumes that in 2020 depression will have a prevalence equal to one in six individuals. Studies of neuroanatomy have highlighted structural alterations in the hippocampus, striatal nuclei and prefrontal cortex in patients with mood disorders. This alteration in depressed patients is closely related to the secretion of neurotrophic factors, in particular there is a reduction in BDNF (Brain Derived Neurotrophic Factor).

**Objectives:**

The objective of this study is to demonstrate which treatments are effective in reducing depressive symptoms that allow the increase of BDNF and consequently the structural homostaticity of the brain.

**Methods:**

We have selected data from the literature of the last decade, collected on major search engines such as: Google Scholar, Research Gate, PubMed, Ebsco. Articles collected by selecting the following Keyword: depression, BDNF (Brain Derived Neurotrophic Factor), neuroimaging cognitive behavior therapy.

**Results:**

The results show that in patients treated with a single drug treatment or vagus nerve stimulation, repetitive transcranial magnetic stimulation (Lang et al., 2008) or electroconvulsive therapy had improvements in BDNF levels, although compared to drug treatment there are problems of no responders, no compliance and lack of effectiveness in reducing vulnerability to relapse. In addition, the study has shown that patients treated with cognitive behavioral therapy have reported greater changes in the frontal and temporal cortex reducing both depressive symptoms and the risk of relapse.

**Conclusions:**

Underlines the importance of an integrated approach

